# Legume flour as a natural colouring component in pasta production

**DOI:** 10.1007/s13197-019-04061-5

**Published:** 2019-08-28

**Authors:** Dorota Teterycz, Aldona Sobota, Piotr Zarzycki, Agnieszka Latoch

**Affiliations:** 1 Division of Engineering and Cereals Technology, Department of Plant Food Technology and Gastronomy, University of Life Sciences in Lublin, Skromna 8, 20-704 Lublin, Poland; 2 Department of Meat Technology and Food Quality, University of Life Sciences in Lublin, Skromna 8, 20-704 Lublin, Poland

**Keywords:** Pasta, Legume flour, Colouring component, Green pea, Red lentil, Grass pea, Cooking quality

## Abstract

In recent years, natural food colouring components have been sought. The conducted study presents the possibility of using different legume flours as a colouring component in durum wheat semolina pasta. The impact of legume flours addition on chemical composition, cooking quality, and sensory properties of pasta was also investigated. The pasta was fortified with 0–20% of green pea, red lentil, and grass pea flours and was made using lamination technology. An increase in the amount of the legume flour caused a significant increase in the content of dietary fibre, ash, protein, and essential amino acids, including lysine. The addition of the legume flours changed the colour of the pasta samples. Most suitable colouring component of all the studied legume flours was the red lentil flour. The pasta with 20% addition of red lentil flour had the most intense colouring (ΔE = 11.31), highest level of consumer acceptance and were characterized by acceptable cooking losses (7.47% d.m.), appropriate weight increase index (2.44), and high firmness.

**Electronic supplementary material:**

The online version of this article (10.1007/s13197-019-04061-5) contains supplementary material, which is available to authorized users.

## Introduction

Pasta is one of the most popular cereal product in the world. Its main advantages are versatility, ease of preparation, low price, and long shelf life (Kaur et al. 2013). In recent years, consumption of pasta has been steadily increasing. This creates a possibility to produce new types of pasta, for example enriched with unconventional raw materials.

Lamination technology, next to “extrusion”, is one of the most popular method of the pasta-making. It is also called as “sheet rolling” or “roll pressure stretching” technique (Hou et al. 2010). The lamination technology consists in preparing a dough with a moisture content of 30–40% in a premixer and then rolling it between steel rollers into a sheet. The diameter between consecutive pairs of rolls decreases, which results in thinner sheets of dough up to the desired thickness. The dough sheets are then cut into appropriate shapes and dried in the same way as the pressed pasta. In laminating technology it does not use vacuum, as it is in the case of extruded pasta production (Zardetto and Dalla Rosa 2006).

The basic and most commonly used raw material for pasta making is durum semolina. It is appreciated for its intense yellow colour, high protein content, and high gluten quality (Troccoli et al. 2000). The strong gluten in durum wheat pasta determines its high cooking quality, including low cooking losses, resistance to overcooking, and good firmness and elasticity after cooking (Sissons 2008). Durum semolina pasta contains 10.9–13.5% protein and more minerals and fat than common wheat flour pasta (Sobota et al. 2015). However, wheat protein is not a valuable source of the indispensable amino acids. Lysine is the limiting amino-acid for its biological value (Mogra and Micha 2013). According to many authors, a good way to increase the nutritional value of wheat pasta is the addition of legume flours (Osorio-Díaz et al. 2008; Wood 2009). Legume seeds are an excellent source of protein (16–55%), rich in essential amino-acids, including lysine. The amino-acid composition of legume protein is similar to meat protein (Sparvoli et al. 2015). In addition, legumes are rich in starch (18–55%) (Sparvoli et al. 2015) and dietary fibre (Dodevska et al. 2013). These raw materials contain minerals, especially iron, zinc, and calcium, as well as biologically active components, including polyphenols and folic acid. In addition, legume seeds are cheap raw materials, often referred to as “poor men’s meat” (Sparvoli et al. 2015; Duranti 2006).

A very important factor determining the choice of a product by the consumer is its colour. The brain associates colour with taste and the initial perception of food occurs during the first 90 s of observation and about 75% of the assessment is based on colour (Singh 2006). In addition, different colour of the well-known product arouses curiosity and willingness to taste (Dias et al. 2012). Synthetic dyes are often added to food products to enhance their attractiveness. Nowadays, there is an increasing demand for natural colouring components that are much more readily accepted and gain greater trust among consumers (Kowalska et al. 2017).

Some legume seeds are characterized by specific colour resulting from the presence of a variety of pigments in the cotyledons and seed coats. For example, the content of chlorophyll (21 mg/100 g) determines the green colour of unripe pea seeds, the carotenoids in red lentils (5–28 mg/100 g) give the seeds their characteristic red–orange colour, and the intense yellow colour of grass pea seeds is the result of the content of lutein (5.68 µg/g) and β-carotene (0.21 µg/g) (Hanh et al. 2016; Zhang et al. 2015; Grela et al. 1999). Therefore, the seed of these legumes can be used not only for fortifying the nutritional value of pasta, but also as natural colouring components determining their attractive colour. There are no studies on the possibility of using legume seeds as natural colouring components in the production of pasta. It is purposeful and justified to determine the share of the additive that allows to obtain an attractive colour of products. It is also important to investigate that the product’s colour is durable and does not change during cooking.

## Materials and methods

### Raw materials

The materials used in this study were semolina durum (Jula Malom, Kunszállás, Hungary) and three legume flours of different colours: green pea flour (P) (FPH Paulas, Ltd., Kalisz, Poland), red lentil flour (RL) (Niedźwiady Mill, Kalisz, Poland), and grass pea flour (GP) (EKORAB, Staszów, Poland).

### Chemical analysis

The chemical composition of the pasta samples and raw materials was determined in accordance with AACC methods (2000). Crude protein content (AACC, Method 46-08) minerals content determined as ash (AACC, Method 08-01) and moisture content (AACC, Method 44-15A were examined. The content of amino acids in selected samples was determined using the AAA 400 analyser (Ingos, Czech Republic). An enzymatic method was used to investigate the content of total dietary fibre (TDF) and its fractions (soluble—SDF and insoluble—IDF) in the pasta and raw materials (AACC 32-05, AACC 32-21, AOAC 991.43, and AOAC 985.29) (AACC 2000; AOAC, 1990).

### Pasta making

The pasta was produced in a long-cut form (Tagliatelle) using the lamination technology. The model of the experiment is presented in Table 1. The dough was prepared by mixing durum semolina with legume flours in a planetary mixer. During stirring in the mixer, water at a temperature of approximately 30 °C was gradually added. The dough was mixed for 3 min, kneaded, and left to rest for 15 min. An Atlas 180 pasta machine (Marcato S.R.L, Campodarsego, Italy) was used to sheet and calibrate the thickness of the dough sheets. The dough thickness was reduced at 4 passages of rollers with a gradually decreasing gap of 4.8, 3.3, 1.9, and 1.5 mm, respectively. The dough sheets were cut into strands of 7 mm wide and 500 mm long and initially dried in a stream of warm air (40 °C) for 2 min. The initially dried pasta strands were formed into nests and placed on the sieves. The nests were dried in pasta static dryers (ESS, type 4C, La Parmigiana S.R.L., Italy) for 7 h. The temperature range was 55–35 °C and relative air humidity was changed from 75% to 55%.

**Table 1:** Model of experiment

Pasta sample	Semolina	Green pea flour (%)	Red lentil flour (%)	Grass pea flour (%)	Water (ml/1000 g of sample)
CON	100				200
P5	95	5			200
P10	90	10			200
P15	85	15			200
P20	80	20			190
RL5	95		5		200
RL10	90		10		200
RL15	85		15		200
RL20	80		20		190
GP5	95			5	200
GP10	90			10	200
GP15	85			15	200
GP20	80			20	190

Flours from: *P* green pea, *RL* red lentil, *GP* grass pea

### Cooking properties of pasta

*Minimal cooking time* was determined by removing the pasta samples from the boiling distilled water every 30 s and squeezing it between two glass plates until the white central core of the pasta disappeared.

*Weight increase index (A)* of the products was determined as:

$$ {\text{A}} = {\text{W}}/{\text{W}}_{0} $$

In this equation W and W_0_ were the weight of cooked and uncooked pasta (g), respectively.

*Cooking loss* was determined by assaying the content of total solids in the liquid after cooking (AACC, Method 44-15A).

### The colour of cooked and raw pasta

The parameters of raw and cooked pasta were determined with the reflective method using a spherical spectrophotometer 8200 (X-Rate, Inc. USA). They were assessed using a standard light source (D65) and a standard colorimetric observer with a 10° visual field. A 12.3 mm diameter hole was used for the measurement. Colour coordinates (L*, a*, b*) were determined using the CIE system. The spectrophotometer was calibrated with white and black standard plates. Changes in the colour of the pasta as a result of addition of the legume flours (∆E) were determined relative to the CON sample (raw and cooked) as:$\Delta E = \sqrt {\left( {L_{2}^{*} - L_{1}^{*} } \right)^{2} + \left( {a_{2}^{*} - a_{1}^{*} } \right)^{2} + \left( {b_{2}^{*} - b_{1}^{*} } \right)^{2} }$,where L_2_*, a_2_*, b_2_* are colour parameters of control sample taken as reference, and L_1_*, a_1_*, b_1_* referred to colour parameters of pasta samples with legume flours.

### Firmness of pasta

A sample of 50 g of pasta was boiled in 600 ml of water for 8 min, drained off for 5 min and cooled for 5 min. The cooked, single strand of pasta was cut on a textured basis. The maximum force required (firmness) to cut the cooked pasta samples (single strand) was determined using a TA-XT Plus texture analyser (Stable Micro System, Godalming, England). Pasta strands were cut with a tooth moving at the speed of 1 mm s^−1^ with a straight steel blade (0.3 mm). On the basis of the trial, the maximum force required for cutting the product [N] was determined.

### Organoleptic analysis

The organoleptic analysis of the cooked pasta was made by a 12-person consumer panel (20–25 years old). Pasta samples was cooked in distilled water for prescribed minimal cooking time and presented on the white plates. Organoleptic characteristics, such as appearance, colour, taste, smell, hardness, adhesiveness and springness were evaluated on a scale of 1 to 5, where 5 was the maximum value of the study parameters. An average note was calculated from all the assessments.

### Statistical analysis

The chemical analyses and assessment of cooking properties were carried out with three replications. Colour parameters were determined in twenty replications, and firmness was evaluated in eight replications for each sample. Mean values were calculated. The significance of differences among the results were determined using the Duncan test (*P* < 0.05). The statistical analysis of the results was performed using the program SAS 9.1.3. (SAS Institute Inc., Cary, NC, USA).

## Results and discussion

The introduction up to 20% of the legume flours: green pea (P), red lentil (RL), and grass pea (GP) to durum pasta guaranteed a high quality of the products. In the samples with the addition of the legume flours an increase in the ash content was noted (Table 2). The ash content was 0.76% in the semolina; in the case of the legume flours, it was higher and ranged from 2.45 to 2.97% (Table 2). According to Jahreis et al. (2016), the ash content in legumes (green pea, lupine) was within the range of 3.1–4.3%.

**Table 2:** Chemical composition of raw materials and pasta samples

Raw materials	Moisture ± SD	Ash ± SD	Protein ± SD	IDF ± SD	SDF ± SD	TDF ± SD
	(%)	(% d.m.)				
Semolina durum	9.26 ± 0.26	0.76 ± 0.01	13.00 ± 0.82	1.62 ± 0.02	1.82 ± 0.06	3.44 ± 0.04
P	7.86 ± 0.06	2.65 ± 0.02	24.50 ± 1.58	11.77 ± 0.33	4.14 ± 0.28	15.91 ± 0.05
RL	10.31 ± 0.04	2.45 ± 0.04	26.62 ± 1.67	13.22 ± 1.07	4.07 ± 0.08	17.29 ± 0.99
GP	7.85 ± 0.09	2.97 ± 0.01	30.23 ± 1.95	11.55 ± 0.17	4.63 ± 0.06	16.18 ± 0.04
*Pasta samples*						
CON	11.99^a^ ± 0,08	0.76^h^ ± 0,02	13.04^g^ ± 0.82	1.75^h^ ± 0.06	1.20^f^ ± 0.04	2.95^h^ ± 0.10
P5	10.08^c^ ± 0.17	0.84^gh^ ± 0.06	13.63^fg^ ± 0.86	1.91^gh^ ± 0.03	1.82^de^ ± 0.13	3.73^g^ ± 0.09
P10	9.88^cde^ ± 0.19	1.00^e^ ± 0.01	14.17^efd^ ± 0.89	2.36^f^ ± 0.11	2.05^bcd^ ± 0.21	4.41^ef^ ± 0.32
P15	9.76^de^ ± 0.16	1.02^e^ ± 0.02	14.54^cde^ ± 0.78	3.29^cd^ ± 0.12	2.14^abcd^ ± 0.01	5.43^bc^ ± 0.12
P20	9.45^f^ ± 0.09	1.25^a^ ± 0.01	15.58^ab^ ± 0.99	3.64^ab^ ± 0.41	2.21^a^ ± 0.14	5.85^ab^ ± 0.54
RL5	10.09^c^ ± 0.05	0.83^gh^ ± 0.02	13.71^efg^ ± 0.86	2.15^fg^ ± 0.05	1.93^de^ ± 0.06	4.08^fg^ ± 0.11
RL10	10.33^b^ ± 0.10	0.92^f^ ± 0.02	14.47^cdef^ ± 0.91	2.70^e^ ± 0.22	2.01^dc^ ± 0.17	4.71^e^ ± 0.05
RL15	9.87^cde^ ± 0.11	1.12^bc^ ± 0.01	15.21^bc^ ± 0.96	3.27^cd^ ± 0.01	2.18^abcd^ ± 0.31	5.45^bc^ ± 0.29
RL20	9.74^de^ ± 0.11	1.08^cd^ ± 0.02	15.89^ab^ ± 1.00	3.92^a^ ± 0.19	2.29^ab^ ± 0.07	6.21^a^ ± 0.27
GP5	9.88^cde^ ± 0.11	0.88^fg^ ± 0.05	13.72^efg^ ± 0.87	1.93^gh^ ± 0.04	1.71^e^ ± 0.19	3.64^g^ ± 0.16
GP10	9.84^de^ ± 0.05	1.01^e^ ± 0.02	14.63^cd^ ± 0.92	2.68^e^ ± 0.14	2.17^abcd^ ± 0.11	4.85^ed^ ± 0.03
GP15	9.99^cd^ ± 0.15	1.05^de^ ± 0.04	15.69^ab^ ± 0.99	3.01^d^ ± 0.19	2.26^abc^ ± 0.08	5.27^cd^ ± 0.27
GP20	9.68^e^ ± 0.21	1.13^bc^ ± 0.02	16.29^a^ ± 1.03	3.59^bc^ ± 0.32	2.39^a^ ± 0.06	5.98^a^ ± 0.39

*CON* control sample (semolina durum pasta), *P* green pea flour, *RL* red lentil flour, *GP* grass pea flour, *SD* standard deviation for three determinations

The different letters (a–h) within the same column indicate statistically significant differences (*P* < 0.05) between the results

The content of dietary fibre (TDF), including soluble (SDF) and insoluble fractions (IDF), in the pasta also increased with the addition of the legume flours (Table 2). The 20% addition of the legume flours to the pasta samples (P, RL, and GP) caused an approximately 2-fold increase in the content of TDF. It was related to its high content in the legume flours (15.91–17.29% d.m.) (Table 2). The highest content of insoluble fibre fractions was recorded for samples RL20 and P20 (3.92 and 3.64% d.m., respectively). The content of the soluble fibre fraction in all the analysed pasta samples was lower than that in IDF and ranged from 1.20% d.m. (CON) to 2.39% d.m. (GP20).

Studies showed that legume flours contain more protein than semolina durum. The investigated legume flours were characterized by a protein content of 24.5–30.23% d.m., while it was only 13% d.m. in the semolina (Table 2). Therefore, the addition of the legume raw material caused a substantial increase in the protein content in the pasta samples. The protein content in the analysed products with 20% addition of the green pea, red lentil, and grass pea flours was 15.58%, 15.89%, and 16.29%, respectively. Similar content of protein (15.9%) in pasta with 20% addition of chickpea flour was noted by Osorio-Díaz et al. (2008).

Amino acids are divided into the following groups: essential and nonessential. Legume proteins are low in sulphur-containing amino-acids (cysteine, methionine). On the other hand, the amounts of an essential amino acid such as lysine are greater than in wheat (Nadathur et al. 2016). Wheat protein supplements deficiencies of sulphuric amino acids in leguminous protein. On the other hand, the protein of leguminous seeds provides lysine, which is missing in wheat. Legume seeds may contain from 1.2 mg/g lysine in chickpea to 2.19 mg/g of lysine in cowpea (Singh et al. 2010). In the pasta with the addition of the legume flours (15%), there was a significant increase in the content of essential amino acids such as lysine (60–88%), threonine (10–33%), and isoleucine (1–22%) (Table 3). The highest content of lysine (4.60 mg/g) was recorded in sample with 15% addition of red lentil flour (RL15). According to Wood (2009) in pasta with a 15% chickpea flour addition, lysine content is lower and amounts to 3.6 mg/g. A significant increase in leucine and phenylalanine was noted only in the case of the pasta with the red lentil (RL) and grass pea (GP) flour addition. In samples with the 15% addition of these legume components, the contents of leucine, phenylalanine, and threonine were higher compared to pasta enriched with navy bean and pinto bean (Bahnassey et al. 1986). On the other hand, the content of methionine and tryptophan in the sample fortified with legume flour (P, RL and GP) were reduced by 6–9% and 9–35%, respectively (Table 3). Compared to the CON sample (semolina durum pasta), higher contents of arginine, valine, and aspartic acids were noted in the pasta with the addition of the all legumes flour (P, RL, GP) (Table 3). Bahnassey et al. (1986) noted a similar trend. In the amino acid composition in pasta samples enriched with 10–15% addition of legume flour (navy bean and pinto bean), there was a significant increase in the content of alanine, arginine, aspartic acid, and glycine.

**Table 3:** Amino acid composition of selected pasta samples (mg/g)

Amino acid	Pasta sample			
	CON	P15	RL15	GP15
*Nonessential*				
Glutamic acid	44.43^c^ ± 0.05	37.79^d^ ± 0.04	45.15^a^ ± 0.06	44.66^b^ ± 0.03
Proline	16.32^a^ ± 0.01	13.41^d^ ± 0.01	15.86^b^ ± 0.01	15.66^c^ ± 0.01
Aspartic acid	5.37^d^ ± 0.03	7.60^c^ ± 0.02	8.99^a^ ± 0.03	8.87^b^ ± 0.03
Serine	5.74^c^ ± 0.01	5.59^d^ ± 0.03	6.97^a^ ± 0.02	6.73^b^ ± 0.01
Glycine	3.62^c^ ± 0.03	3.89^b^ ± 0.03	4.60^a^ ± 0.03	4.55^a^ ± 0.03
Tyrosine	2.37^c^ ± 0.02	2.42^c^ ± 0.03	2.64^b^ ± 0.03	2.86^a^ ± 0.01
*Essential*				
Leucine	8.12^c^ ± 0.02	8.05^d^ ± 0.01	9.73^a^ ± 0.02	9.43^b^ ± 0.03
Phenylalanine	6.09^c^ ± 0.01	6.12^c^ ± 0.02	7.44^a^ ± 0.01	7.06^b^ ± 0.02
Valine	4.67^c^ ± 0.03	4.73^c^ ± 0.01	5.70^a^ ± 0.03	5.53^b^ ± 0.03
Tryptophan	4.45^a^ ± 0.01	2.89^d^ ± 0.02	4.06^b^ ± 0.02	3.85^c^ ± 0.01
Threonine	3.01^d^ ± 0.02	3.31^c^ ± 0.03	4.01^a^ ± 0.02	3.93^b^ ± 0.02
Methionine	2.78^a^ ± 0.01	2.63^b^ ± 0.02	2.37^d^ ± 0.02	2.47^c^ ± 0.02

*CON* control sample (semolina durum pasta), *P* green pea flour, *RL* red lentil flour, *GP* grass pea flour, *SD* standard deviation for three determinations

The different letters (a–d) within the same row indicate statistically significant differences (*P* < 0.05) between the results

Dick and Youngs (1988) argue that high quality pasta is characterized by a threefold weight increase after cooking. For wheat extruded pasta weight increase index to reach the level of 2.14–4.14 (Sobota and Skwira 2009). In presented study significantly lower water uptake was noted for the pasta with the addition of legume flours compared to the semolina durum pasta (CON). The index of weight increase for cooked products was between 2.24 (GP10, GP20) and 2.61 (CON) (Table 4). This effect can be caused by weakening of the gluten matrix due to the addition of legumes flour which does not contain gluten. Doxastakis et al. (2007) and Petitot et al. (2010) also observed a decrease in water absorption during cooking, when spaghetti was enriched with lupine or faba bean flour. Rosa-Sibakov et al. (2016) related this tendency with the higher swelling of starch granules. According to these authors, water was able to penetrate more easily in legume-enriched pasta allowing faster starch gelatinization and which reduced water absorption during cooking.

**Table 4:** Cooking quality and firmness of pasta samples

Pasta sample	Minimal cooking time	Weight increase index ± SD	Cooking loss ± SD	Firmness ± SD
	(min)		(%)	(N)
CON	7.5	2.61^a^ ± 0.06	5.02^f^ ± 0.13	3.32^d^ ± 0.12
P5	7.5	2.46^b^ ± 0.04	5.71^e^ ± 0.14	4.15^b^ ± 0.23
P10	8	2.46^b^ ± 0.01	6.42^d^ ± 0.03	4.13^b^ ± 0.12
P15	8	2.48^b^ ± 0.05	7.48^b^ ± 0.22	4.05^b^ ± 0.07
P20	8	2.49^d^ ± 0.04	7.58^b^ ± 0.11	2.86^e^ ± 0.28
RL5	8	2.4^b^ ± 0.07	6.63^d^ ± 0.27	3.31^d^ ± 0.12
RL10	8	2.32^c^ ± 0.08	7.01^c^ ± 0.18	3.16^d^ ± 0.18
RL15	8	2.32^c^ ± 0.05	6.98^c^ ± 0.52	3.87^c^ ± 0.20
RL20	8	2.44^b^ ± 0.02	7.47^b^ ± 0.04	5.02^a^ ± 0.38
GP5	7.5	2.35^c^ ± 0.03	5.25^f^ ± 0.14	3.35^d^ ± 0.20
GP10	8.5	2.24^d^ ± 0.02	7.13^c^ ± 0.45	1.93^f^ ± 0.12
GP15	8.5	2.25^d^ ± 0.03	7.46^b^ ± 0.19	2.05^f^ ± 0.08
GP20	8.5	2.24^d^ ± 0.04	8.23^a^ ± 0.28	2.00^f^ ± 0.06

*CON* control sample (semolina durum pasta), *P* green pea flour, *RL* red lentil flour, *GP* grass pea flour, *SD* standard deviation for three determinations

The different letters (a–f) within the same column indicate statistically significant differences (*P* < 0.05) between the results

One of the major parameters of pasta cooking quality is cooking losses. Pasta of satisfactory quality should not lose more than 8% d.m. (Dick and Youngs 1988). The laminated pasta enriched with 5–20% of the legume flours (P, RL, GP) was characterized by relatively low cooking losses (5.02–8.23%), but still higher than CON. The highest passage into the cooking water observed for the pasta with the 20% addition of the legume flours, but the cooking loss exceeded 8% only for one of the analysed samples (GP20). Similar cooking losses (4.8–5.8%) were noted by Ahmad et al. (2018) in extruded pasta fortified with 5–25% detoxified matri flour (grass pea). In the case pasta-like sheets enriched with a protein isolate and dietary fibre from yellow pea, Muneer et al. (2018) noted cooking losses between 41.2% and 45.8%. As regards of traditional wheat pasta, an increase in the gluten protein content is associated with lower cooking losses (Sobota and Zarzycki 2013), but substitution of gluten proteins (gliadins and glutenins) by legume protein increase cooking losses. This is attributed to dilution of the gluten network and weakening of its overall structure (Laleg et al. 2017). Rosa-Sibakov et al. (2016) emphasize that legume proteins (mainly globulins and albumins) do not form an elastic network such as gluten proteins. Poor elasticity of the protein matrix may cause passage of dry matter into cooking water. In addition, the higher levels of dietary fibres in legume flours than in semolina durum (15.91–17.29% d.m. vs. 3.44% d.m.) (Table 2) may also weaken the gluten matrix and increase dry matter losses (Tables 2 and 4).

Firmness is a very important quality factor of pasta. The highest firmness was exhibited by the pasta with the lentil flour addition (RL20), while the lowest firmness was noted for the pasta with the grass pea flour (GP10-20) (Table 4). The study has shown that firmness depends on the type and amount of the legume flour inclusion to the products. Fortification of the pasta with 5–15% of the green pea flour (P) and 15–20% of the red lentil flour (RL) significantly increased the firmness. A reverse tendency was noted in the case of samples enriched with 10–20% of the grass pea flour (GP). Sissons et al. (2005), Zhao et al. (2005), and Petitot et al. (2010) reported that pasta enriched with legume flour (faba bean, green and yellow pea, lentil, and chickpea) was characterized by higher hardness and firmness than durum wheat pasta. The authors suggested that the tendency could be related to the higher protein content in these products. A significant influence on the texture parameters may be exerted by water absorption during cooking. Lower water absorption recorded in the case of high protein pasta (with addition of legume components such as lupine, protein isolate and faba bean flour) may be a factor determining higher firmness and hardness of the products (Rosa-Sibakov et al. 2016; Petitot et al. 2010). A longer cooking time of pasta contributes to higher moisture content in products (Sobota et al. 2015) and may cause a decrease in pasta firmness and hardness. Dziki et al. (2013) observed that the addition of soybean flour decreased pasta firmness.

The legume flour was used as a natural colouring component in the pasta. Colour parameters (L*, a*, b*) of raw and cooked pasta changed significantly with the addition of the legume flours (Table 5). The lightness (L* value) of all raw and cooked samples of the pasta decreased as the legume components increased. This result is in agreement with studies conducted by Zhao et al. (2005), Wood (2009), and Petitot et al. (2010). The darker colour of the legume-supplemented pasta may be attributed to the higher content of ash and the specific colour of the legume flour. Parameter a* (green–red) for each raw pasta sample increased with the addition of the legume flours. The uncooked pasta fortified with the green pea flour (P) became less yellow (b*), but samples with the red lentil flour and the grass pea flour were characterized by more intensive yellow colour (b*). As a result of cooking, the lightness (L*), redness (a*), and yellowness (b*) of the pasta was reduced. Similarly, Petitot et al. (2010) noted a decrease in redness and yellowness, but found that brightness (L*) of legume-fortified pasta increased after cooking. A significant increase in parameter a* was noted for the cooked pasta sample with 5–20% addition of red lentil (RL) and 20% addition of green pea flour (P). An opposite tendency was observed for samples with the grass pea flour (GP). Pasta samples fortified with 10–20% of the grass pea flour (GP) characterised significant more green colour compared to the control sample pasta (CON).

**Table 5:** Colour parameters for raw and cooked pasta samples

Pasta sample	Raw pasta				Cooked pasta			
	L* ± SD	a* ± SD	b* ± SD	ΔE	L* ± SD	a* ± SD	b* ± SD	ΔE
CON	74.64^a^ ± 1.81	− 0.17^i^ ± 1.70	21.01^f^ ± 1.59	–	68.95^a^ ± 1.00	− 2.08^fg^ ± 0.12	9.27^c^ ± 0.93	–
P5	73.38^b^ ± 2.58	− 0.18^i^ ± 1.54	18.80^h^ ± 0.97	2.54	67.79^b^ ± 1.44	− 2.15^gh^ ± 0.14	8.53^d^ ± 1.43	1.38
P10	72.66^c^ ± 1.87	0.03^h^ ± 0.08	19.66^g^ ± 0.90	2.40	67.46^bc^ ± 1.73	− 2.05^fg^ ± 0.12	10.39^ab^ ± 1.03	1.86
P15	70.81^d^ ± 2.53	0.45^g^ ± 0.31	19.68^g^ ± 3.24	4.10	66.94^bcd^ ± 0.80	− 2.07^fg^ ± 0.14	10.48^ab^ ± 0.74	2.35
P20	68.94^f^ ± 2.05	0.55^gf^ ± 0.14	23.94^d^ ± 1.31	6.45	65.92^def^ ± 1.48	− 1.86^e^ ± 0.18	9.94^b^ ± 1.11	3.73
RL5	74.42^ab^ ± 1.96	2.47^d^ ± 0.37	19.11^gh^ ± 0.68	3.26	64.80^g^ ± 2.39	− 1.64^d^ ± 0.15	7.50^e^ ± 0.92	2.87
RL10	71.06^d^ ± 2.10	4.58^c^ ± 0.35	21.01^f^ ± 0.74	5.94	65.57^gef^ ± 2.96	− 1.40^c^ ± 0.17	8.65^cd^ ± 0.97	3.51
RL15	70.24^de^ ± 1.89	6.78^b^ ± 0.35	22.33^e^ ± 1.07	8.33	65.32^gf^ ± 1.86	− 1.00^b^ ± 0.25	8.85^cd^ ± 1.38	3.77
RL20	69.50^ef^ ± 1.68	9.08^a^ ± 0.56	25.01^c^ ± 1.03	11.31	66.42^cde^ ± 1.90	− 0.90^a^ ± 0.29	10.39^ab^ ± 1.54	3.84
GP5	73.67^abc^ ± 2.38	− 0.10^hi^ ± 0.14	18.69^h^ ± 0.84	2.51	68.95^a^ ± 1.39	− 2.01^f^ ± 0.18	9.05^cd^ ± 0.91	0.23
GP10	70.11^def^ ± 1.44	− 0.02^hi^ ± 0.13	23.22^d^ ± 0.83	5.04	67.25^bc^ ± 2.00	− 2.19^h^ ± 0.14	9.36^cd^ ± 1.19	1.71
GP15	67.73^g^ ± 1.92	0.70^f^ ± 0.19	26.80^b^ ± 0.80	9.06	66.65^cde^ ± 1.71	− 2.21^h^ ± 0.19	10.08^ab^ ± 1.09	2.44
GP20	66.82^g^ ± 1.76	0.89^e^ ± 0.29	27.87^a^ ± 1.86	10.46	65.63^efg^ ± 1.19	− 2.33^i^ ± 0.14	10.78^a^ ± 1.18	3.66

*CON* control sample (semolina durum pasta), *P* green pea flour, *RL* red lentil flour, *GP* grass pea flour, *SD* standard deviation for three determinations

The different letters (a–i) within the same column indicate statistically significant differences (*P* < 0.05) between the results

One of the parameters in the assessment of pasta colour is the L* a* b* difference (ΔE). ΔE is an equally weighted combination of the coordinate (L*, a*, b*) differences. This parameter significantly increased with the addition of the legume flours. The most intense colour was caused by the addition of the red lentil flour (RL), which is why this flour seems to be the best of the studied colouring components for pasta production. ∆E for the uncooked RL20 pasta was 11.31, whereas this value for the cooked product was much smaller and amounted to 3.84. In all the tested pasta samples, the colour difference (ΔE) decreased after cooking. A similar trend was recorded by Wood (2009) for pasta with addition of chickpea flour. The smallest change in colour as a result of pasta cooking and, consequently, the highest colour fastness was recorded for the samples fortified with the green pea flour.

A very important factor determining the quality of a food product is the balance between its nutritional value and consumer acceptability. Therefore, consumers’ assessment is very important before the product is placed on the market. The pasta samples were evaluated on a five-point scale by a random group of 30 consumers for their appearance, colour, taste, smell, hardness, adhesiveness, and springiness (Table 6). On the basis of individual assessments, an average score for each sample was calculated. The highest score for the fortified samples was noted for the GP5 sample, probably due to its high similarity to the CON sample. Nevertheless, the small differences in the evaluation values (4.13–4.27) indicate that the pasta samples with the red lentil flour (RL) are the most acceptable products.

**Table 6:** Sensory analysis

Pasta sample	Appearance	Colour	Taste	Smell	Hardness	Adhesiveness	Springness	Average score
CON	4.8	4.6	4.6	4.7	4.8	4.8	4.4	4.65
P5	4.4	4.2	4.2	4.6	4.8	4.8	4.2	4.45
P10	4.1	4.2	4.2	4.6	4.3	4.8	4.1	4.33
P15	3.6	3.8	3.8	4.2	4.2	4.2	3.5	3.90
P20	3.9	4.2	3.9	4.4	4.5	4.2	3.5	4.08
RL5	4	4.3	4	4.4	4.3	4.3	4.2	4.21
RL10	4	3.9	4.4	4.7	4.3	4.4	4.2	4.27
RL15	3.5	3.6	4.4	4.4	4.6	4.6	4.2	4.19
RL20	3.2	3.8	4.2	4.6	4.4	4.5	4.2	4.13
GP5	4.2	4.6	4.5	4.6	4.5	4.6	4.4	4.49
GP10	3.4	3.9	4.1	4.5	4.5	4.6	4	4.14
GP15	2.9	3.6	3.6	4.1	4.6	4.2	3.6	3.80
GP20	2.4	3.6	3.8	3.9	4.5	4.3	3.7	3.74

*CON* control sample (semolina durum pasta), *P* green pea flour, *RL* red lentil flour, *GP* grass pea flour

## Conclusion

The present studies show the possibility of using legumes flour to change the colour and improve the amino acid composition of pasta products. The red lentil flour (RL) was the best colouring component. The addition of legume flour increases significantly the protein and dietary fibre content in pasta (TDF, IDF, SDF). The content of lysine, i.e. a limiting amino acid in wheat products, increased by 60–88% in the pasta samples fortified with the 15% addition of the legume flours. The pasta with the legume flours was characterized by acceptable values of weight increase and cooking losses during cooking. Incorporation selected legume flour e.g. red lentil flour to the semolina durum pasta may have a positive impact on intensity of pasta colour and its consumer acceptance.

## Electronic supplementary material

Below is the link to the electronic supplementary material.

Supplementary material 1 (DOCX 268 kb)
