# Supplementary figures and images for: Legume flour as a natural colouring component in pasta production
Source: J Food Sci Technol. 2019 Aug 28;57(1):301–9. doi: 10.1007/s13197-019-04061-5 (PMC6952492; doi:10.1007/s13197-019-04061-5)

Appendix 1


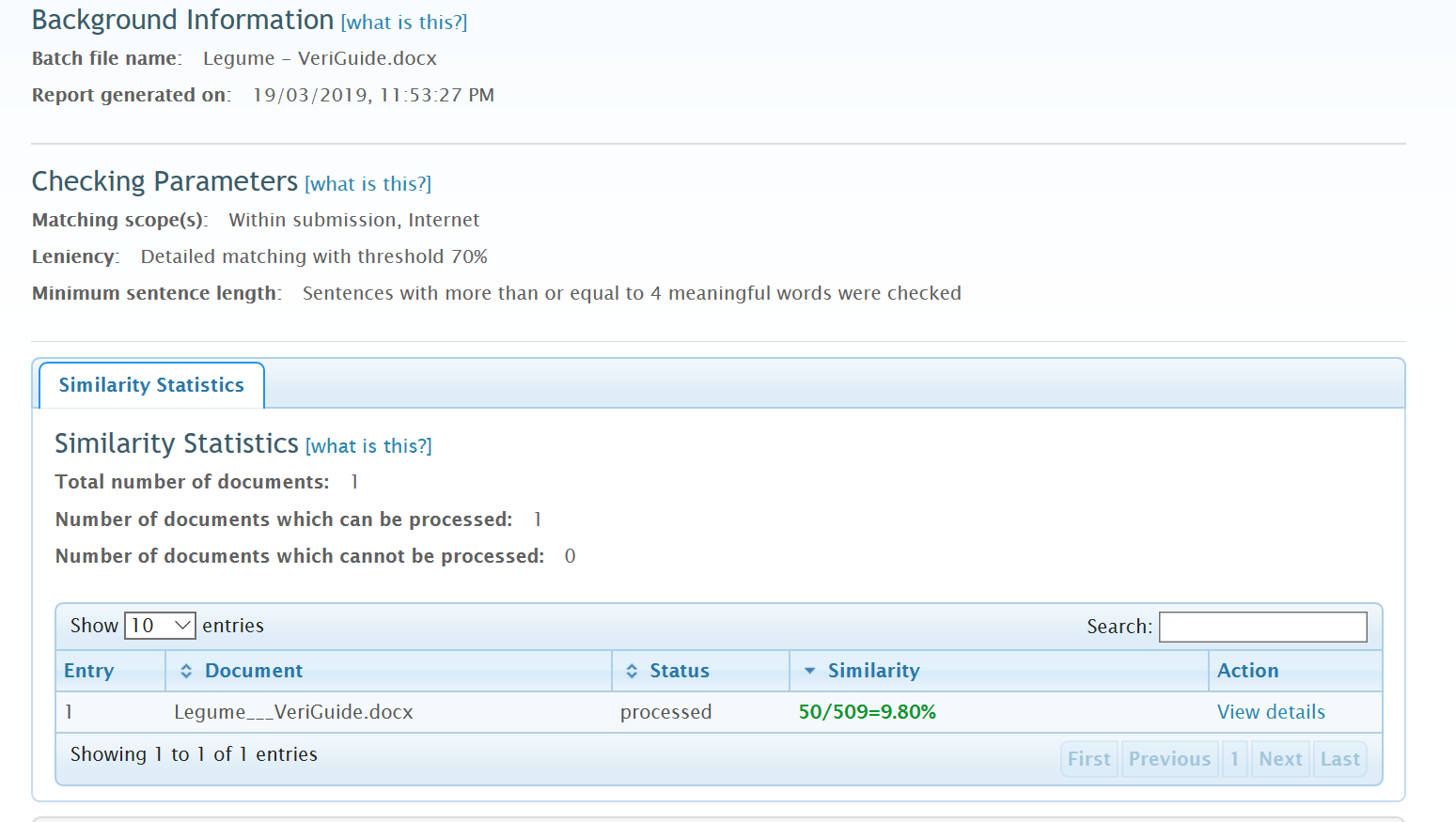

Supplement: Supplementary file 1 — Supplementary material 1 (DOCX 268 kb) [file 13197_2019_4061_MOESM1_ESM.docx]
